# Acupuncture for Chronic Pain-Related Depression: A Systematic Review and Meta-Analysis

**DOI:** 10.1155/2021/6617075

**Published:** 2021-02-22

**Authors:** Jianyu You, Haiyan Li, Dingyi Xie, Rixin Chen, Mingren Chen

**Affiliations:** ^1^Jiangxi University of Traditional Chinese Medicine, Nanchang, China; ^2^The Affiliated Hospital of Jiangxi University of Traditional Chinese Medicine, Nanchang, China

## Abstract

**Objective:**

The aim of this systematic review was to summarize and evaluate the existing evidence on the effectiveness and safety of acupuncture in relieving chronic pain-related depression (CPRD).

**Methods:**

We searched seven online databases to identify eligible randomized controlled trials (RCTs) of acupuncture for CPRD published before September 2020. We included studies that used acupuncture as the intervention group, with or without a control group, and the control group was treated with conventional drugs. Meta-analysis was performed using RevMan 5.3 software. For outcomes, assessments were performed using the Hamilton Depression Scale (HAMD), Visual Analogue Scale (VAS), and adverse events.

**Results:**

Eight studies involving 636 participants were identified and included in the meta-analysis. The results showed that single acupuncture treatment and drug treatment have the same effect in improving the HAMD score (MD = −0.14, 95% CI = [−0.88, 0.59], *P* = 0.71) and alleviating the VAS score (MD = −0.42, 95% CI = [−1.10, −0.27], *P* = 0.23), but acupuncture treatment is safer (OR = 0.03, 95% CI = [0.01, 0.21], *P* = 0.0003). In addition, acupuncture combined with drugs (control group) is more beneficial than single-drug treatment in improving the HAMD score (MD = −2.95, 95% CI = [−3.55, −2.36], *P* < 0.00001) and alleviating the VAS score (MD = −1.06, 95% CI = [−1.65, −0.47], *P* = 0.0004).

**Conclusion:**

Acupuncture is an effective and safe treatment for CPRD, and acupuncture combined with drug therapy is more effective than single-drug therapy. Nevertheless, the conclusions were limited due to the low quality and a small number of included studies.

## 1. Introduction

Chronic pain (CP) is defined as pain that continues beyond 3 months. Internationally, no less than 20% of adults (18–65 years) and more than 33% of older adults (>65 years) suffer from CP [1]. In addition, the latest estimates claim that CP affects approximately 1.5 billion people worldwide, and these numbers are rising steadily [2]. In the United States, CP is thought to affect more than 116 million adults, which is higher than the combined prevalence of heart disease, diabetes, and cancer [2]. Due to the high prevalence of CP, it has brought a heavy economic burden to the healthcare system and society. In European countries, pain caused by chronic low back pain and musculoskeletal diseases has been proven to cost up to 2% of the gross domestic product (GDP) [3], while in the United States, the annual cost of CP treatment can be as high as $635 billion [4]. In addition, CP can not only lead to a reduction in the quality of life of patients but also has a huge negative impact on the mental health of patients. Studies have shown that CP is often accompanied by a wide range of mental disorders, of which depression is one of the more common comorbidities [5].

Depression, the fourth leading cause of disability worldwide, is defined as a psychological problem characterized by negative mood, hopelessness, and despair [6, 7]. In most developed countries, the lifetime prevalence of major depression is 16.2% [8]. Additionally, it is estimated that depression and its related diseases will become the main contributor to the global burden of disease by 2030 [9]. According to reports, the average prevalence of major depression in CP patients is about 50% [9], and the anxiety or major depression of patients with pain increases 2.5–10 times compared with the general population [10, 11]. This striking level of comorbidity suggests that there may be a bidirectional relationship between CP and depression. That is, CP can cause negative emotions such as anxiety and depression, and negative emotions can also lead to and accelerate pain. The existence of pain has a significant negative impact on the clinical management of depression, making the treatment of depression more complicated, and depression has a similar impact on the clinical management of pain [12, 13].

In this study, CPRD was defined as a type of comorbid depression associated with CP, with clinical manifestations of CP and depression. Depression can be caused by pain, or depression itself exists and is accompanied by pain symptoms, both of which are considered in this study. Although the pathogenesis of CPRD is still unclear, in primary care, the guidelines suggest CPRD is primarily managed with antidepressants and painkillers [14–16]. These therapies are associated with low remission rates and high dropout rates [17], and the use of these drugs is also limited by serious side effects, such as dependence, gastrointestinal reactions, and allergies [18]. In addition, psychotherapy is considered to be a safe and effective treatment method, but the clinical application of psychotherapy is limited by the lack of qualified therapists. Therefore, it is an urgent research question to find an alternative treatment that can alleviate relieve both depressive symptoms and coexisting pain [5].

Acupuncture, an alternative nondrug treatment, is an important part of traditional Chinese medicine and involves the use of thin needles to stimulate specific acupoints on the human body. In China, acupuncture has been widely used to treat diseases for at least 3000 years. In addition, acupuncture is also one of the most popular alternative therapies in the world. Modern research has shown that acupuncture can stimulate the neuroendocrine of the body by stimulating specific points on the body. Therefore, acupuncture has a satisfactory therapeutic effect on diseases involving neuroendocrine pathological changes (such as chronic pain, menopause, depression, and insomnia) [19, 20], with a very rare occurrence of adverse events.

Although the benefits of acupuncture treatment of CP and depression have been widely reported [21–24], a recent meta-analysis on acupuncture for chronic pain with depression found that acupuncture is efficient and safe therapy [25]. However, the experimental group of the study consisted of single acupuncture and acupuncture combined with other therapies, and the study did not perform a subgroup analysis of the interventions of the experimental group. These factors affected the accuracy of the conclusions of the study. Therefore, we conducted this study to evaluate the existing evidence from RCTs to separately evaluate the effectiveness and safety of acupuncture and acupuncture-related comprehensive therapies for CPRD.

## 2. Methods

This systematic review was performed in accordance with the Preferred Reporting Items for Systematic Reviews and Meta-Analyses (PRISMA) Statement and was registered at PROSPERO (number CRD42019146188) [26].

### 2.1. Search Strategy

We searched digital databases for RCTs which evaluated the effectiveness of acupuncture for CPRD, including Embase, PubMed, Cochrane Library, WanFang, CNKI, VIP, and the Chinese SinoMed Database (up to September 2020). The keywords used for the search consist of three parts: chronic pain (e.g., musculoskeletal pain, back pain), depression (e.g., depression, affective disorder, affective symptoms, mood), and acupuncture (e.g., acupuncture, electroacupuncture needling, acupoint). The complete search terms are shown in Supplementary Materials. References of related articles were manually checked for potential eligible RCTs for inclusion.

### 2.2. Inclusion and Exclusion Criteria

Studies were included if the following situations were met: (1) types of studies: Only RCTs of acupuncture therapy for CPRD were included. RCTs were published in English or Chinese; (2) types of participants: Participants met the diagnosis of depression and chronic pain at the same time; (3) type of intervention: The only experimental treatments allowed are manual acupuncture or electroacupuncture alone, or either of these combined with the control group (drugs); (4) types of control groups: The control group should be conventional drug therapy, and the method, dosage, and course of treatment were reported in detail. There are no restrictions on the drugs used here, including western medicine and Chinese herbal medicine, and they may also include drugs that are no longer used in some countries (for example, Deanxit); (5) types of outcome measures: primary outcomes were Hamilton Depression Scale (HAMD) and Visual Analogue Scale (VAS), the secondary outcome was adverse events; (6) Full text should be available.

The exclusion situations included the following: (1) Non-RCTs; (2) RCTs that compared different kinds of acupuncture; (3) duplicate studies; (4) case reports; (5) animal experiments.

### 2.3. Data Extraction

Two authors (Jianyu You, Haiyan Li) independently extracted relevant data from studies that met the inclusion criteria. Key information included the first author, publication year, sample size, baseline characteristics of participants, intervention, major outcomes (measured at the end of treatment), and adverse events. If there is any uncertainty, we will resolve it through discussion or consultation with the corresponding author (Rixin Chen).

### 2.4. Quality Assessment

The quality and risk of bias (ROB) of the included RCTs were independently evaluated by two authors (Jianyu You, Haiyan Li) using the Cochrane risk of bias assessment tool [27]. The contents include (1) random sequence generation; (2) allocation concealment; (3) blinding of participants and personnel; (4) blinding of outcome assessment; (5) incomplete outcome data; (6) selective reporting; (7) other sources of bias. For each item, ROB was graded as high, unclear, or low. Discrepancies were resolved through discussion with the corresponding author (Rixin Chen).

### 2.5. Statistical Analysis

Data analysis was performed using Reviewer Manager software. For continuous data (HAMD and VAS), we estimated the combined mean difference (MD) with 95% confidence intervals (CI); for the dichotomous data (adverse events), we calculated the combined odds ratio (OR) with 95% CI. Heterogeneity was evaluated by Higgins *I*^2^ test and chi-square test. When *I*^2^ ≤ 50%, *P* ≥ 0.10, and the fixed effect model was applied; otherwise, the random effect model was used, and subgroup analysis was performed to explore heterogeneity. Subgroup analyses were carried out according to the different intervention measures. If the number of included studies was insufficient, we did not assess publication bias.

## 3. Results

### 3.1. Literature Search Results

**Figure 1:**
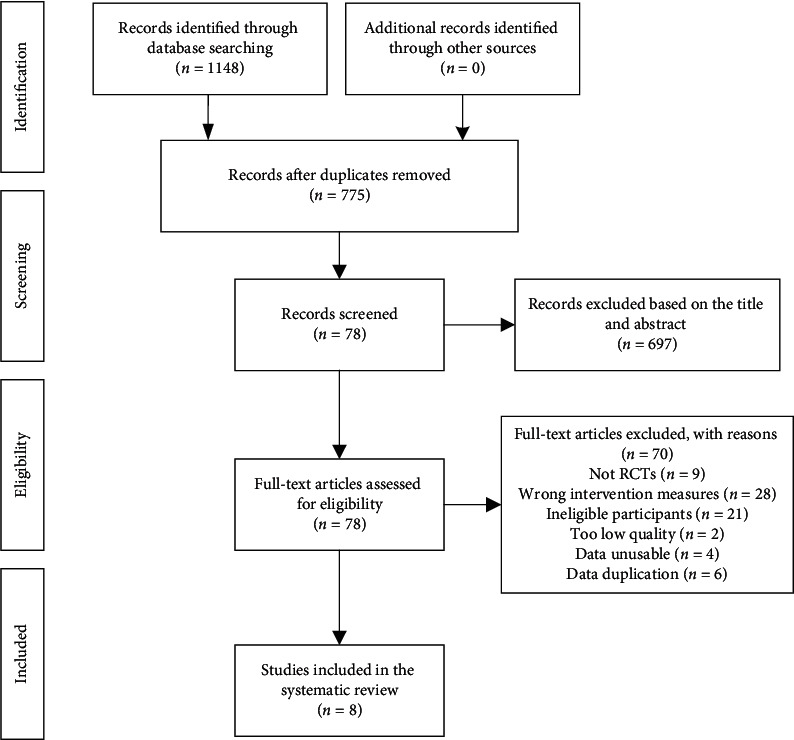
Flow diagram of the study.

A total of 1148 studies were retrieved from all initial searches. 775 studies remained after we excluded 373 duplicates, and 697 studies were eliminated based on the title and abstract. Then, the eligibility of the remaining 78 studies was evaluated by scanning the full text. Finally, 8 RCTs [28–35] met the inclusion criteria and were included in the systematic review. The screening process is shown in Figure 1.

### 3.2. Basic Information of Included Studies

We included a total of 8 RCTs, involving 636 participants, including 316 in the experimental group and 320 in the control group. All studies were conducted in China, including one article [29] published in English and seven published in Chinese. The sample size of included studies ranges from 40 to 128. There were three studies [28, 29, 33] that compared single acupuncture with drugs, and the remaining five studies compared acupuncture-combined drugs with drugs. One study [28] used electroacupuncture, and the other seven studies used manual acupuncture. Characteristics of included studies are shown in Table 1.

### 3.3. Quality Assessment

**Figure 2:**
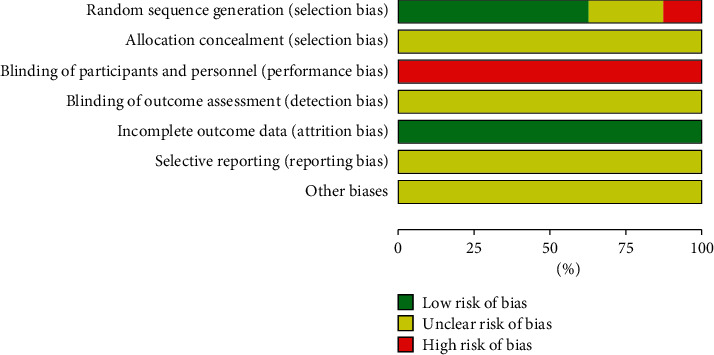
Risk of bias graph: review authors' judgements about each risk of bias item presented as percentages across all included studies.

**Figure 3:**
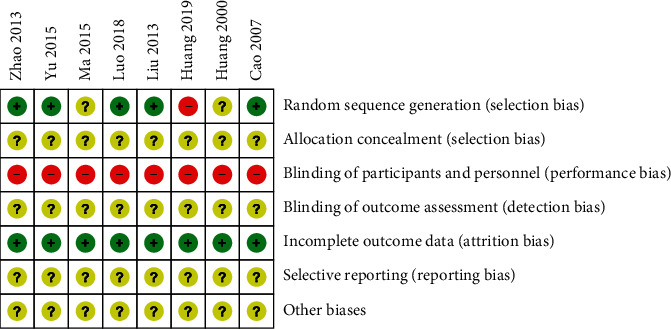
Risk of bias summary: review authors' judgements about each risk of bias item for each included study.

Among all the included 8 RCTs, five studies [29–31, 33, 34] used a random number table to generate random sequences for grouping and were assessed as low ROB. One study [35] was randomized according to the order of admission of participants and was assessed as having a high ROB. The remaining studies did not mention the method or details of random sequence generation and were judged as unclear ROB. No studies mentioned the details of the use of allocation concealment, and all studies were assessed as unclear ROB. Due to the characteristics of acupuncture therapy, it is difficult to perform blinding operations. Therefore, all studies were judged to have a high ROB in blinding. No study reported the blinding details about outcome assessment, and all studies were considered to have an unclear ROB. All studies had no loss of outcome data and were considered to have a low ROB. Since all included studies have no published protocol or trial registration records, the reporting bias of all included studies was considered to have an unclear ROB. All studies were judged as unclear ROB due to a lack of clear evidence to show the existence of other biases. The ROB summary is presented in Figures 2 and 3.

### 3.4. HAMD Score

**Figure 4:**
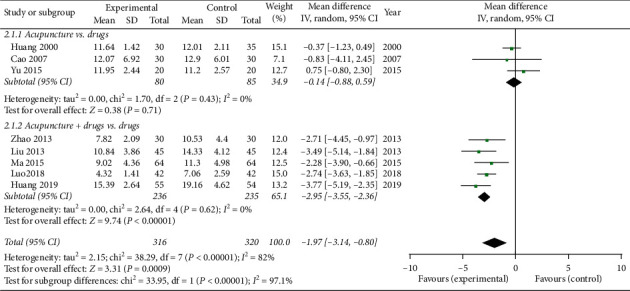
Meta-analysis for the HAMD score of acupuncture versus the control group.

All studies evaluated the severity of depression by using the HAMD score. Three studies [28, 29, 33] compared acupuncture with drugs, and five studies [30–32, 34, 35] compared acupuncture combination drugs with drugs alone. Due to the high heterogeneity (*P* < 0.00001, *I*^2^ = 82%), we used a random-effects model. The results show that experimental groups could further relieve depression compared with control groups (MD = −1.97, 95% CI = [−3.14, −0.80], *P* < 0.00001). Subgroup analysis also showed that acupuncture combination drugs are statistically significantly better than single drugs (MD = −2.95, 95% CI = [−3.55, −2.36], *P* < 0.00001). However, acupuncture only was not statistically superior to drugs alone (MD = −0.14, 95% CI [−0.88, 0.59], *P* = 0.71) (Figure 4).

### 3.5. VAS Score

**Figure 5:**
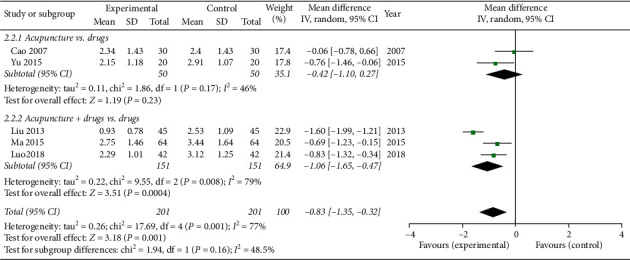
Meta-analysis for the VAS score of acupuncture versus the control group.

Five studies [29, 30, 32–34] evaluated pain intensity by using the VAS score. Two studies [29, 33] compared acupuncture with drugs, aND three studies [30, 32, 34] compared acupuncture combination drugs with drugs alone. The random-effects model was used due to the heterogeneity in the data (*P* = 0.001, *I*^2^ = 77%). The results show that experimental groups could further relieve pain compared with the control group (MD = −0.83, 95% CI = [−1.35, −0.32], *P* = 0.001). Subgroup analysis also showed that acupuncture combination drugs are statistically significantly better than drugs (MD = −1.06, 95% CI = [−1.65, −0.47], *P* = 0.0004). However, there was no statistically significant difference between acupuncture and oral drugs (MD = −0.42, 95% CI = [−1.10, −0.27], *P*= 0.23, heterogeneity: *P* = 0.17, *I*^2^ = 46%) (Figure 5).

### 3.6. Adverse Events

**Figure 6:**
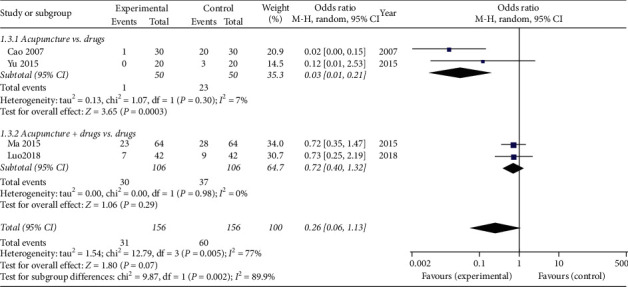
Meta-analysis for adverse events of acupuncture versus the control group.

Six studies [28, 29, 32–35] reported the occurrence of adverse events, of which only four studies [29, 32–34] reported the exact number of adverse events. Two studies [29, 33] compared acupuncture with drugs, and two studies [32, 34] compared acupuncture combination drugs with drugs alone. Obvious heterogeneity was found among these RCTs (*P* = 0.005, *I*^2^ = 77%), and the random-effects model showed no statistical difference in adverse events between the experimental group and the control group (OR = 0.26, 95% CI = [0.06, 1.13], *P* = 0.07). In addition, subgroup analysis also showed the same results between acupuncture combination drugs and drugs (OR = 0.72, 95% CI = [0.40, 1.32], *P* = 0.29). However, single acupuncture treatment has a lower incidence of adverse events compared to oral drugs (OR = 0.03, 95% CI = [0.01, 0.21], *P* = 0.0003) (Figure 6).

### 3.7. Publication Bias

Since the number of included studies did not exceed 10, funnel plots were not used to measure publication bias.

## 4. Discussion

Depression and pain are the most common psychological and physical symptoms in primary care, respectively. In addition, depression and pain often coexist (30%–50% cooccurrence) [36]. Pain has a negative impact on the prognosis and treatment of depression and vice versa. There is a significant correlation between the severity of pain and the degree of depression [37]. Although the specific pathogenesis of depression and pain is still unclear, current experimental evidence suggests that the pathophysiological processes of depression and pain overlap in many aspects. For example, the brain structures involved in pain and depression shared neural circuits, and neurochemicals play an important role in the formation of pain and depression [6, 37, 38].

Acupuncture is a part of Traditional Chinese Medicine (TCM), which has the advantages of easy operation, safety, economy, and reliable efficacy [39]. At present, acupuncture has been widely used in the clinical treatment of various diseases in many countries around the world, among which chronic pain and depression are common diseases treated by acupuncture [23]. The mechanism of acupuncture analgesia is quite complex, involving the entire nervous system from the periphery to the center. Modern research has shown that acupuncture analgesia is related to the interaction of a variety of biologically active molecules in the pain process, including neurotransmitters, inflammatory mediators, cell signaling molecules and neuropeptides, etc. [39–41]. At the same time, relevant depression research also affirmed the antidepressant effects of acupuncture [42]. Recent studies have provided laboratory-based evidence that acupuncture treatment can increase the expression of 5-HT1A receptors in the cortex, hippocampus, thalamus, and hypothalamus, as well as the expression of 5-HT1B in the cortex and thalamus. Therefore, acupuncture can effectively relieve depression symptoms [19, 43].

In the present study, we included 8 RCTs to compare the effects of acupuncture and oral drugs, as well as acupuncture-combined oral drugs and oral drugs. With respect to improving the depression symptoms, the HAMD score was used to indicate the intensity of depression. All RCTs evaluated HAMD scores using the same scale, so we reported the results using the mean difference (MD) of HAMD. Our pooled analysis indicated that acupuncture-combined oral drugs were more effective than single oral drugs. However, there was no statistically significant difference between acupuncture and oral drugs. With respect to reducing pain, the VAS score was used to indicate the intensity of pain. Five RCTs evaluated VAS scores using the same scale, so we reported the results using the mean difference (MD) of VAS. Our pooled analysis indicated that acupuncture combined with oral drugs was more effective than single oral drugs. However, the combined data showed no significant difference between acupuncture and oral drugs. In this study, four RCTs reported relevant adverse events with the exact number. The results showed that there was no significant difference in adverse reactions between the experimental group and the control group. Additionally, the subgroup analysis also showed the same results between acupuncture combination drugs and single drugs. However, single acupuncture treatment has a lower incidence of adverse events compared to oral drugs. Therefore, we can cautiously recommend that acupuncture is a safe treatment for CPRD. Based on the results of our included studies, we suggest that acupuncture is an effective and safe alternative therapy for CPRD.

This systematic review has several limitations. Firstly, the insufficient number of RCTs were included in our systematic review, and most of the RCTs had a relatively small sample size. This limitation may lead to inaccurate research evidence. Secondly, the quality of the included RCTs was not satisfactory. Some studies lack the details of random sequence generation, and no RCTs mentioned the use of allocation concealment and blind details, which may lead to imprecise evidence in our study. Thirdly, there was considerable heterogeneity in our study. Subgroup analyses were used to explore the source of heterogeneity. Lastly, all RCTs were conducted in China, which may lead to publication bias and affect the validity and reliability of this systematic review.

## 5. Conclusions

The results of our current systematic review and meta-analysis show that compared with drug treatment, single acupuncture treatment has the same effect in reducing pain and relieving symptoms of depression in patients with CPRD, but the incidence of adverse reactions of acupuncture treatment is smaller. In addition, acupuncture combined with drug therapy has a better effect than a single drug. However, due to the insufficient number of included studies, low methodological quality, and heterogeneity of results, further studies using large- and high-quality samples are needed to confirm the role of acupuncture for CPRD.

## Figures and Tables

**Table 1:** Characteristics of included studies.

Study	Sample size	Mean age (SD)	Sex (male/female)	Diagnosis	Interventions' group	Control group	Treatment period	Outcomes
Huang and Luo [28]	65	30.39 ± 7.01	25/40	Depression: CCMD-2-R CP : CD	EA	Medicine (amitriptyline)	T: once a day, six times per week for 6 weeks, 20 min C: once a day for 6 weeks	HAMD, AE

Cao et al. [29]	60	NR	23/37	CD	MA	Medicine (Deanxit)	T: once a day, five times per week for 4 weeks, 30 min C: twice a day for the first 10 days and once a day for the next 18 days for 4 weeks	HAMD, VAS, AE

Liu et al. [30]	90	T: 47 ± 8 C: 48 ± 8	T: 15/30 C: 16/29	Depression: CCMD-3 CP : CD	MA + C	Medicine (SSRI antidepressants)	T: once every two days for 4 weeks, 30 min; drug treatment was the same as the control group C: once a day for 4 weeks	HAMD, VAS

Zhao et al. [31]	60	NR	T: 12/18 C: 11/19	Depression: CD CP : ICHD	MA + C	Medicine (diclofenac sodium)	T: twice a day, 6 times a week for 30 days, 30 minutes; drug treatment was the same as the control group C: once a day for 30 days	HAMD

Ma et al. [32]	128	T: 39.93 ± 12.93 C: 38.69 ± 14.19	T: 27/37 C: 29/35	Depression: ICD-10 CP : CD	MA + C	Medicine (duloxetine + benzodiazepines)	T: five times per week for 8 weeks, 20 min; drug treatment was the same as the control group C: duloxetine once a day for 8 weeks, benzodiazepines were used according to the needs of the disease	HAMD, VAS, AE

Yu et al. [33]	40	T: 41 ± 8 C: 40 ± 7	T: 6/14 C: 8/12	Depression: CD CP : CCMD-3	MA	Medicine (Deanxit)	T: once a day, six times a week for 8 weeks, 50 min : twice a day for 8 weeks	HAMD, VAS, AE

Luo et al. [34]	84	T: 57.15 ± 11.26 C: 57.39 ± 11.58	T: 22/20 C: 23 /19	CD	MA + C	Medicine (Chinese herbal medicine)	T: once a day for 4 weeks, 30 min; drug treatment was the same as the control group C: one dose a day for 4 weeks	HAMD, VAS, AE

Huang et al. [35]	109	T: 45.36 ± 2.78 C: 45.72 ± 2.79	T: 24/31 C: 22 /32	Chinese diagnostic criteria	MA + C	Medicine (Deanxit or fluoxetine + olanzapine)	T: five times a week for 2 months, 30 min; drug treatment was the same as the control group C: moderate depression: Deanxit twice a day for two months; severe depression: fluoxetine once a day for two months, olanzapine was used according to the needs of the disease	HAMD, AE

AE: adverse events; C: control group; CCMD: Chinese Classification of Mental Disorders; CD: clinical diagnosis; EA: electroacupuncture; HAMD: Hamilton Depression Scale; ICD: International Classification of Diseases; ICHD: International Classification of Headache Disorders; MA: manual acupuncture; NR: not reported; T: therapy group; VAS: Visual Analogue Scale.

## Data Availability

All data generated or analyzed during this study are included within this published article and its supplementary information files.

## Supplementary Materials

Supplementary Materials Supplemental materials explain the complete search process. Our search process consists of three parts: chronic pain (e.g., musculoskeletal pain and back pain), depression (e.g., depression and affective disorder), and acupuncture (e.g., acupuncture and electroacupuncture).
