# Supplementary material for: Acupuncture for Chronic Pain-Related Depression: A Systematic Review and Meta-Analysis
Source: Pain Res Manag. 2021 Feb 22;2021:6617075. doi: 10.1155/2021/6617075 (PMC7925064; doi:10.1155/2021/6617075)
Supplement: Supplementary Materials — Supplemental materials explain the complete search process. Our search process consists of three parts: chronic pain (e.g., musculoskeletal pain and back pain), depression (e.g., depression and affective disorder), and acupuncture (e.g., acupuncture and electroacupuncture). [file 6617075.f1.pdf]

Supplementary Material

Literature search strategy

The search algorithm was constructed as follows:

#1 Mesh term: (acupuncture OR electroacupuncture OR needling OR acupoint)

#2 Mesh term: (depression OR affective disorder OR affective symptoms OR mood)

#3 Mesh term: (pain OR chronic pain OR long-lasting pain OR long-term pain OR intermittent pain OR persistent pain OR neuropathic pain OR neuralgia OR intractable pain OR nociceptive pain OR whiplash injuries OR rheumatoid OR musculoskeletal pain OR myalgia OR fibromyalgia OR myofascial pain OR headache OR migraine OR muscle OR tendon OR ligament OR bone OR cartilage OR spine OR cervical vertebrae OR thoracic vertebrae OR lumbar vertebrae OR sacrum OR coccyx OR intervertebral disc OR ankylosing spondylitis OR arthritis OR arthralgia OR polyarthritis OR joint OR osteoarthritis OR rheumatoid arthritis OR neck pain OR shoulder pain OR back pain OR backache OR low back pain OR pelvic girdle pain OR upper extremity pain OR arm pain OR elbow pain OR forearm pain OR hand pain OR lower extremity pain OR hip pain OR knee pain OR heel pain OR foot pain OR metatarsalgia OR leg pain OR lumbosacral region pain OR sacrococcygeal region pain)

#4: 1 AND 2 AND 3

Note: When searching the Chinese databases, we used Chinese key words and translated them in this article.
